# Short report: association between self-reported COVID-19 experience and contemptuous beliefs about pandemic management among German citizens and healthcare professionals

**DOI:** 10.1093/pubmed/fdaf144

**Published:** 2025-11-08

**Authors:** Odette Wegwarth, Ralph Hertwig

**Affiliations:** Heisenberg Chair for Medical Risk Literacy & Evidence-Based Decisions, Charité – Universitätsmedizin Berlin, Berlin, Charitéplatz 1, 10117 Berlin, Germany; Center for Adaptive Rationality, Max Planck Institute for Human Development, Berlin, Lentzeallee 94, 14195 Berlin, Germany; Center for Adaptive Rationality, Max Planck Institute for Human Development, Berlin, Lentzeallee 94, 14195 Berlin, Germany

**Keywords:** COVID-19 pandemic, description–experience gap, pandemic preparedness, public health communication, risk perception

## Abstract

**Background:**

The Coronavirus disease 2019 (COVID-19) pandemic highlighted the importance of public adherence to pandemic management measures. Contempt for these measures could undermine compliance in future pandemics. This study explored associations between self-reported COVID-19 experiences and contemptuous beliefs about COVID-19 pandemic management.

**Methods:**

A cross-sectional online survey study was conducted in September 2024 with 964 German citizens and 423 healthcare professionals from respondi panels (Cologne, Germany). Respondents reported their attitudes toward eight contemptuous statements regarding COVID-19 pandemic management and their personal COVID-19 experiences, including infection, vaccine side effects, long COVID, and patient care (for professionals). Associations were analyzed using logistic regression and Mann–Whitney U tests.

**Results:**

Citizens with self-reported experience of COVID-19 infections were less likely to hold contemptuous beliefs (OR: 0.58; 95% CI: 0.39–0.85; *P* = .005), while those with experience of vaccine side effects were considerably more likely (OR: 1.50; 95% CI: 1.10–1.92; *P* = .009). Long COVID had no significant effect. Among professionals, not having cared for COVID-19 patients doubled the likelihood of contempt (OR: 2.10; 95% CI: 1.28–3.45; *P* = .003).

**Conclusion:**

Findings suggest that experiential factors may contribute to belief formation—an area with limited empirical attention but potential relevance for addressing societal polarization.

## Introduction

In the aftermath of the Coronavirus disease 2019 (COVID-19) pandemic, few experts are questioning the inevitability of future health crises. A critical examination of prevailing views about the role of science and government in managing pandemics is therefore essential. Most government bodies and scientific communities maintain that the mandated and recommended health measures were effective. Critics argue, however, that this is a selective interpretation and that several measures lacked evidence or were disproportionate to the actual risk.^1^ The tension between these viewpoints is likely to influence not only how the public perceive and interpret health risks during future crises, but also their trust in governmental responses and the scientific community.

Cognitive science suggests that risk perception and interpretation are shaped by two modes of learning: personal experience and description. People often perceive their own experience as more authoritative and tangible than descriptions of risk. Experience can thereby exert a stronger influence on beliefs, attitudes, and behaviors than descriptive information (i.e. scientific information)—a phenomenon known as the description–experience gap.^2–8^ However, experience is not bias-free. If experience samples are too small, the true risk distribution in the environment is obscured, resulting in a biased risk assessment.^9^

Previous studies have investigated the influence of direct and indirect experiences with COVID-19 on risk perceptions^10^ and protective behaviours,^11^^,^^12^ yet none have examined whether such experiences also influence the endorsement of contemptuous beliefs about pandemic management. To determine whether the description–experience gap influences contemptuous beliefs about pandemic management, we investigated whether COVID-19 experiences were associated with differences in agreement with publicly discussed contemptuous statements about pandemic management in a national sample of German citizens and healthcare professionals.

## Methods

A cross-sectional national sample of 964 German residents and 423 German healthcare professionals aged ≥18 years—drawn from well-established, probability-based internet panels maintained by respondi (Cologne, Germany)—completed an online study in September 2024. Only respondents who indicated having had at least one experience of three possible COVID-19-related events—infection, vaccine-related side effects, or long COVID—were included in the study. Due to the lack of official statistics on demographic distributions related to these experiences in Germany, a quota sampling method could not be established. However, the sample of German residents is approximately representative of the general German population in terms of gender, age, and educational attainment (Table 1). Respondents saw eight contemptuous statements about the pandemic response, originally used in a large cross-cultural study by Sprengholz *et al*.^13^ and inspired by a report in the German newspaper Die ZEIT (e.g. “The COVID-19 measures were a pretext to restrict civil liberties,” “Evidence shows that most COVID-19 measures did not work,” and “The risk of a COVID-19 infection was low compared to the risks of vaccination side effects”; Cronbach’s α = 0.92; see Supplementary material for full item list). Respondents rated agreement with the eight contemptuous statements on a five-point Likert scale from “I absolutely agree” to “I absolutely disagree” (Supplementary material). They also reported on the frequency and severity of personal COVID-19-related health experiences, including infection, vaccine side effects, and long COVID (Supplementary material). To assess the influence of proxy experiences, healthcare professionals were included in the study and asked about their involvement in COVID-19 patient care, including intensive care.

**Table 1:** Demographic characteristics of the survey samples of German citizens and health professionals.

	German citizens (*N* = 964)	Health professionals (*N* = 423)
	No. (% | % German population)^a^,^b^	No. (%)^d^
Female	481 (49.9 | 50.4)	341 (80.6)^c^
Age (years)		
18–29	154 (16.0 | 15.3)	48 (11.3)
30–39	178 (18.5 | 18.9)	101 (23.9)
40–49	163 (16.9 | 14.2)	103 (24.3)
50–59	222 (23.0 | 18.8)	117 (27.7)
≥60	247 (25.6 | 33.8)	54 (12.8)^c^
Education		
No school leaving certificate	20 (2.1 | 4.0)	3 (0.7)
Lower secondary education	246 (25.5 | 26.6)	33 (7.8)
Intermediate secondary education	296 (30.7 | 27.5)	182 (43.0)
Tertiary entrance qualification	198 (20.5 | 21.5)	133 (31.4)
University degree (e.g. BA, MA)	204 (21.2 | 20.4)	72 (17.0)

^a^Percentages are rounded and may not total 100.

^b^National census data are retrieved from Destatis, OCED/Education GPD, and Wikipedia.

^c^Age group ≥60 years is restricted to 65 years in the sample of professionals due to German retirement regulations.

^d^A total of 67% of the sample were nurses, who still tend to be women in Germany.

The primary endpoint was the difference in agreement with contemptuous statements between respondents with and without COVID-19-related experiences. As a continuous analysis, we computed a mean agreement score by averaging Likert-scale responses across the eight contemptuous belief statements. We then calculated Spearman’s rank correlations (ρ/rho) between this score and ranges of COVID-19-related variables, including: infection status (binary), number of infections (continuous), severity of COVID-19 infection (continuous), severity of vaccine side effects (continuous), Long COVID status (binary), severity of Long COVID (continuous), COVID-related patient care experience (binary), and intensive care experience (binary). Correlations were interpreted as meaningful only if ρ/rho > 0.10, indicating at least a small effect. For results with ρ/rho-values below 0.10, we retained the null hypothesis, even if the corresponding *P*-value was statistically significant. To complement the continuous analysis, we also conducted a binary endorsement analysis, where “I somewhat agree” or “I absolutely agree” responses were coded as endorsements. Participants who endorsed ≥50% of the eight contemptuous statements were classified as showing high endorsement. Associations with COVID-19-related experiences, controlling for demographics, were examined using logistic regression. The effects of experiences on individual contemptuous statements were examined using two-sided Mann–Whitney U tests, with *P*-values adjusted for multiple comparisons using the Holm–Bonferroni method (α = 0.05). As part of this correction procedure, *P*-values were ordered from smallest to largest and compared sequentially to the following adjusted significance thresholds: 0.0063, 0.0071, 0.0083, 0.0100, 0.0125, 0.0167, 0.025, and 0.05. Significance was retained only for *P*-values equal or smaller than their respective threshold. Analyses were conducted using SPSS version 29.0.1.1 (IBM). The study, approved by the Max Planck Institute for Human Development IRB (A2024–16), obtained online informed consent from all respondents.

## Results

Among citizens (*N* = 964), continuous analysis showed that COVID-19 infection (ρ = 0.14, *P* < .001) and infection severity (ρ = 0.12, *P* < .001) were associated with lower mean agreement scores, while vaccine side effects were associated with higher mean agreement scores (ρ = −0.21, p < .001). The binary analysis yielded largely similar patterns. Citizens who reported having had at least one COVID-19 infection (*n* = 815) were only half as likely to highly endorse contemptuous statements (i.e. agreeing with ≥50% of statements) than those reporting no infection (odds ratio [OR]: 0.58; 95% confidence interval [CI]: 0.39–0.85; *P* = .005). In contrast, citizens who reported having experienced vaccine side effects requiring medical attention (*n* = 310) expressed high endorsement nearly twice as often as respondents without that experience (OR: 1.50; 95% CI: 1.10–1.92; *P* = .009). Long COVID or number of infections had no influence on endorsement.

**Figure 1:**
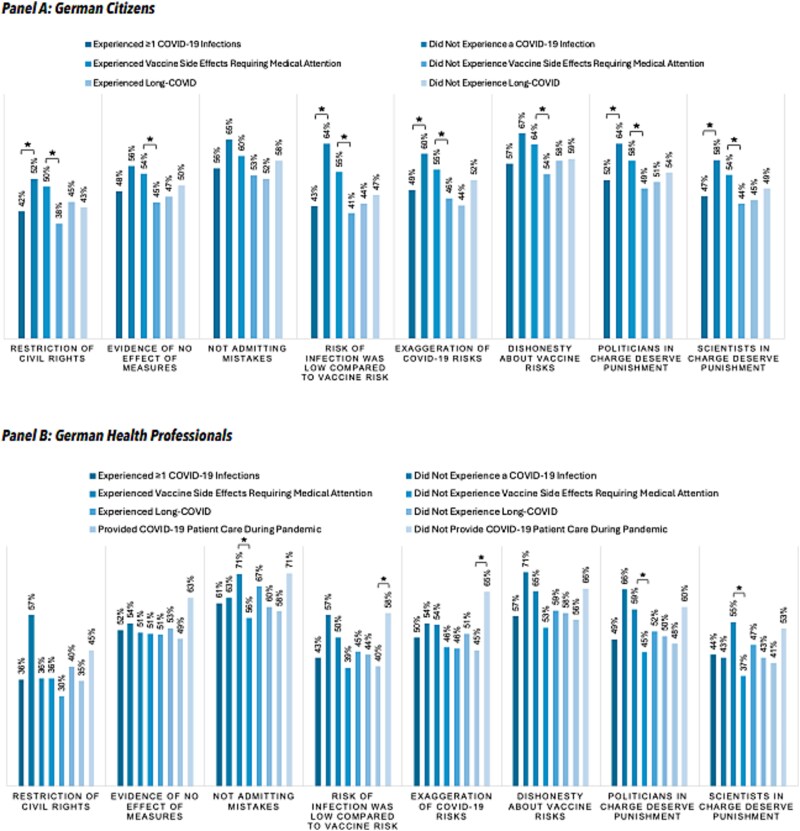
Proportion of endorsement for each contemptuous statement among citizens and healthcare professionals. Statistical significance was assessed using two-sided Mann–Whitney U tests, with *P*-values corrected for multiple comparisons using the holm–Bonferroni method. Significant differences are marked with an asterisk (*).

For health professionals (*N* = 423), continuous analysis showed that involvement in COVID-19-patient care was associated with lower mean agreement scores (ρ = 0.17, *P* < .001), while vaccine side effects were associated with higher scores (ρ = −0.25, *P* < .001). The binary analysis confirmed a significant association only for COVID-19 patient care: health professionals who had provided such care during the pandemic (*n* = 326) were less than half as likely to endorse four or more of the contemptuous statements compared to those without this experience (*n* = 97; OR = 0.47, 95% CI: 0.29–0.78, *P* = .003). Other experiences showed no significant effects. Figure 1 presents the effects of experiential factors on endorsement for each contemptuous belief individually. Figure 1 presents the effects of respondents’ various experiences on their endorsement to each individual contemptuous statement.

Gender and education did not influence endorsement in either sample. Age mattered only among citizens: Respondents in the COVID-19 risk group (i.e. aged ≥60 years) were less likely to express high endorsement (OR: 0.69; 95% CI: 0.51–0.94; *P* = .020).

## Discussion

### Main findings

In our samples, personal or proxy experiences with COVID-19 were associated with endorsement of contemptuous statements about pandemic measures in distinct ways: Experiencing COVID-19 infection and providing patient care during the pandemic were linked to reduced endorsement, whereas experiencing vaccine side effects was linked to increased endorsement. However, not all experiences weighted the same. Factors like long COVID, number of infections, or providing intensive care for COVID-19 patients had no significant impact. Proxy experiences via patient care outweighed most personal experiences among health care professionals, suggesting the interplay between vicarious and personal experiences in shaping beliefs.

### What is already know on this topic

Previous research has highlighted the role of factors such as political ideology and social media^14^ in influencing public attitudes and beliefs during the pandemic. However, empirical work on the influence of direct or vicarious health-related experiences—particularly in the context of contemptuous or polarizing beliefs—has been scarce.

### What this study adds

While the observed associations in our study were small to moderate, our findings contribute to understanding how experiential factors may influence public opinion dynamics and the formation of contemptuous beliefs, and suggest potential avenues for preventive communication strategies in future health crises.

### Limitations

Our study has limitations. First, we cannot exclude potential sources of bias, including non-response, self-selection, social desirability, and ideological bias. Second, generalizability is limited by the inclusion of only the German population and individuals with at least one COVID-19-related experience. Third, while reverse causality cannot be ruled out, individuals with contemptuous beliefs were likely less inclined to get vaccinated, making a reverse explanation for overreported side effects less plausible.

## Conclusion

Notwithstanding these limitations, our findings suggest that experience can play a role in belief formation and point to potential avenues for preventive action. Such action reaches from a more transparent communication of scientific evidence with regard to the measures taken,^15^ overcoming nontransparent health communication,^16^ and the use of interactive simulations in public health communication. The latter has been shown, for instance, to be more effective than text-based formats in reducing misconceptions about vaccine effectiveness (including COVID-19 vaccines) and opioid misuse,^17–19^ and can be easily implemented in educational apps or online health information platforms. While not tested yet in the context of contemptuous belief formation, they may also be a promising tool to attenuate the risk of experiential distortions in future crises.

## Supplementary Material

STROBE_checklist_cross-sectional_NaCoDe_fdaf144

Supplement_NaCoDe_Wegwarth_JPH-25-0092_R1_fdaf144

## Data Availability

Data are available from the corresponding author upon reasonable request.

## Notes

**Odette Wegwarth**, PhD & professor

**Ralph Hertwig**, PhD & professor
