# Supplementary material for: Short report: association between self-reported COVID-19 experience and contemptuous beliefs about pandemic management among German citizens and healthcare professionals
Source: J Public Health (Oxf). 2025 Nov 8;48(1):332–7. doi: 10.1093/pubmed/fdaf144 (PMC13017043; doi:10.1093/pubmed/fdaf144)
Supplement: Supplement_NaCoDe_Wegwarth_JPH-25-0092_R1_fdaf144 [file supplement_nacode_wegwarth_jph-25-0092_r1_fdaf144.docx]

Supplement

Wegwarth, O. Hertwig, R.: Association Between Self-Reported COVID-19 Experience and Contemptuous Beliefs About Pandemic Management Among German Citizens and Healthcare Professionals

- I. Survey Questions page 2
- II. Proportions of Corona-Related Experiences Among the Lay Sample page 4
- III. Proportions of Corona-Related Experiences Among the Medical Professional Sample page 6

**I. SURVEY QUESTIONS (TRANSLATED FROM GERMAN)**

Derogatory Narratives About Corona Pandemic

1. The COVID-19 measures were a pretext to restrict civil liberties.

(I completely agree, I somewhat agree, I have no opinion on this, I hardly agree, I do not agree at all.)

1. Evidence shows that most COVID-19 measures did not work.

(I completely agree, I somewhat agree, I have no opinion on this, I hardly agree, I do not agree at all.)

1. Science and politics are now portraying the containment measures in a positive light, to avoid admitting any mistakes.

(I completely agree, I somewhat agree, I have no opinion on this, I hardly agree, I do not agree at all.)

1. The risk of a COVID-19 infection was low compared to the risks of vaccination side effects.

(I completely agree, I somewhat agree, I have no opinion on this, I hardly agree, I do not agree at all.)

1. Science and politics exaggerated the severity of the COVID-19 virus during the pandemic.

(I completely agree, I somewhat agree, I have no opinion on this, I hardly agree, I do not agree at all.)

1. Science and politics were not honest about the benefits and risks of the COVID-19 vaccine.

(I completely agree, I somewhat agree, I have no opinion on this, I hardly agree, I do not agree at all.)

1. Politicians should be punished for how they handled the COVID-19 pandemic.

(I completely agree, I somewhat agree, I have no opinion on this, I hardly agree, I do not agree at all.)

1. Scientists who gave advice to the government should be punished for how they handled the COVID-19 pandemic.

(I completely agree, I somewhat agree, I have no opinion on this, I hardly agree, I do not agree at all.)

Corona-related experiences

1. How often have you experienced a COVID-19 infection (criterion: positive test result)?

___ times. [If 0 times, proceed to question 2; if 1 time, proceed to a; if more than 1 time, proceed to 1b]

1a. How did this COVID-19 infection progress? [if 1 time]

- The infection was symptomless.
- Despite symptoms, I was able to recover from the infection independently at home.
- I had to consult a general practitioner/medical specialist due to the infection.
- I had to consult a general practitioner/medical specialist multiple times due to the infection.
- I was admitted to a hospital due to the infection.

1b. You had multiple COVID-19 infections. How did the most recent infection progress? [if more than 1 time]

- The infection was symptomless.
- Despite symptoms, I was able to recover from the infection independently at home.
- I had to consult a general practitioner/medical specialist due to the infection.
- I had to consult a general practitioner/medical specialist multiple times due to the infection.
- I was admitted to a hospital due to the infection.

2. Have you been vaccinated against COVID-19 and if, did you experience any adverse effects from the vaccine, and if so, how did they progress?

- I have not been vaccinated against COVID-19.
- I have not experienced any vaccine-related adverse effects after vaccination.
- I was able to recover from the vaccine-related adverse effects independently at home.
- I had to consult a general practitioner/medical specialist due to the vaccine-related adverse effects.
- I had to consult a general practitioner/medical specialist multiple times due to the vaccine-related adverse effects.
- I was admitted to a hospital due to vaccine-related adverse effects.

3. The term "Long COVID" refers to long-term health impairments following a COVID-19 infection that persist beyond the acute illness phase of four weeks. Have you experienced Long-COVID, and how did it progress?

- I have not experienced Long-COVID.
- I was able to recover from the symptoms of Long COVID independently at home.
- I had to consult a general practitioner/medical specialist due to symptoms of Long COVID.
- I had to consult a general practitioner/medical specialist multiple times due to symptoms of Long COVID.
- I was admitted to a hospital due to symptoms of Long COVID.
- I have experienced Long COVID symptoms for three months or longer, which continue to affect me to this day.

[For individuals in the "medical personnel" occupational group only]

1. Were you actively involved in patient care during the COVID-19 pandemic? Yes/No

a. [If Yes] Were you involved in intensive care of patients with a COVID-19 infection? Yes/No

**II. PROPORTIONS OF CORONA-RELATED EXPERIENCES AMONG THE LAY SAMPLE**

| 1. How often have you experienced a COVID-19 infection (criterion: positive test result)? | | |
| --- | --- | --- |
|  | N | % |
| 0 | 149 | 15.5% |
| 1 | 377 | 39.1% |
| 2 | 245 | 25.4% |
| 3 | 135 | 14.0% |
| 4 | 30 | 3.1% |
| 5 | 9 | 0.9% |
| 6 | 6 | 0.6% |
| 7 | 5 | 0.5% |
| 8 | 3 | 0.3% |
| 9 | 1 | 0.1% |
| 10 | 2 | 0.2% |
| 16 | 1 | 0.1% |
| 20 | 1 | 0.1% |

| 1a. How did this COVID-19 infection progress? [if 1 time] | | | |
| --- | --- | --- | --- |
|  | | N | % |
| Despite symptoms, I was able to recover from the infection independently at home. | | 199 | 20.6% |
| I had to consult a general practitioner/medical specialist due to the infection. | | 106 | 11.0% |
| I had to consult a general practitioner/medical specialist multiple times due to the infection. | | 43 | 4.5% |
| I was admitted to a hospital due to the infection. | | 10 | 1.0% |
| The infection was asymptomatic. | | 19 | 2.0% |
| Exclusion due to question logic |  | 587 | 60.9% |

| 1b. You had multiple COVID-19 infections. How did the most recent infection progress? [if more than 1 time] | | | |
| --- | --- | --- | --- |
|  | | N | % |
| Despite symptoms, I was able to recover from the infection independently at home. | | 230 | 23.9% |
| I had to consult a general practitioner/medical specialist due to the infection. | | 130 | 13.5% |
| I had to specialist multiple times due to the infection. | | 47 | 4.9% |
| I was admitted to a hospital due to the infection | | 14 | 1.5% |
| The infection was asymptomatic. | | 17 | 1.8% |
| Exclusion due to question logic |  | 526 | 54.6% |

| 2. If you have been vaccinated against COVID-19, did you experience any adverse effects from the vaccine, and if so, how did they progress? | | |
| --- | --- | --- |
|  | N | % |
| I was able to recover from the vaccine-related adverse effects independently at home. | 308 | 32.0% |
| I had to consult a general practitioner/medical specialist due to the vaccine-related adverse effects. | 176 | 18.3% |
| I had to consult a general practitioner/medical specialist multiple times due to the vaccine-related adverse effects. | 92 | 9.5% |
| I was admitted to a hospital due to vaccine-related adverse effects. | 42 | 4.4% |
| I have not experienced any vaccine-related adverse effects after vaccination. | 289 | 30.0% |
| I have not been vaccinated against COVID-19. | 57 | 5.9% |

| 3. The term "Long COVID" refers to long-term health impairments following a COVID-19 infection that persist beyond the acute illness phase of four weeks. Have you experienced Long-COVID, and how did it progress? | | |
| --- | --- | --- |
|  | N | % |
| I had to consult a general practitioner/medical specialist multiple times due to symptoms of Long COVID. | 88 | 9.1% |
| I was admitted to a hospital due to symptoms of Long COVID. | 18 | 1.9% |
| I have experienced Long COVID symptoms for three months or longer, which continue to affect me to this day. | 66 | 6.8% |
| I have not experienced Long-COVID. | 792 | 82.2% |

**III. PROPORTIONS OF CORONA-RELATED EXPERIENCES AMONG THE HEALTHCARE PROFESSIONAL SAMPLE**

| 1. How often have you experienced a COVID-19 infection (criterion: positive test result)? | | |
| --- | --- | --- |
|  | N | % |
| 0 | 35 | 8.3% |
| 1 | 133 | 31.4% |
| 2 | 142 | 33.6% |
| 3 | 78 | 18.4% |
| 4 | 20 | 4.7% |
| 5 | 8 | 1.9% |
| 6 | 3 | 0.7% |
| 7 | 1 | 0.2% |
| 8 | 1 | 0.2% |
| 13 | 1 | 0.2% |
| 18 | 1 | 0.2% |

| 1a. How did this COVID-19 infection progress? [if 1 time] | | | |
| --- | --- | --- | --- |
|  | | N | % |
| Despite symptoms, I was able to recover from the infection independently at home. | | 68 | 16.1% |
| I had to consult a general practitioner/medical specialist due to the infection. | | 43 | 10.2% |
| I had to consult a general practitioner/medical specialist multiple times due to the infection. | | 15 | 3.5% |
| I was admitted to a hospital due to the infection. | | 6 | 1.4% |
| The infection was asymptomatic. | | 1 | 0.2% |
| Exclusion due to question logic |  | 290 | 68.6% |

| 1b. You had multiple COVID-19 infections. How did the most recent infection progress? [if more than 1 time] | | | |
| --- | --- | --- | --- |
|  | | N | % |
| Despite symptoms, I was able to recover from the infection independently at home. | | 161 | 38.1% |
| I had to consult a general practitioner/medical specialist due to the infection. | | 54 | 12.8% |
| I had to specialist multiple times due to the infection. | | 26 | 6.1% |
| I was admitted to a hospital due to the infection | | 5 | 1.2% |
| The infection was asymptomatic. | | 9 | 2.1% |
| Exclusion due to question logic |  | 168 | 39.7% |

| 2. If you have been vaccinated against COVID-19, did you experience any adverse effects from the vaccine, and if so, how did they progress? | | |
| --- | --- | --- |
|  | N | % |
| I was able to recover from the vaccine-related adverse effects independently at home. | 164 | 38.8% |
| I had to consult a general practitioner/medical specialist due to the vaccine-related adverse effects. | 65 | 15.4% |
| I had to consult a general practitioner/medical specialist multiple times due to the vaccine-related adverse effects. | 43 | 10.2% |
| I was admitted to a hospital due to vaccine-related adverse effects. | 9 | 2.1% |
| I have not experienced any vaccine-related adverse effects after vaccination. | 125 | 29.6% |
| I have not been vaccinated against COVID-19. | 17 | 4.0% |

| 3. The term "Long COVID" refers to long-term health impairments following a COVID-19 infection that persist beyond the acute illness phase of four weeks. Have you experienced Long-COVID, and how did it progress? | | |
| --- | --- | --- |
|  | N | % |
| I had to consult a general practitioner/medical specialist multiple times due to symptoms of Long COVID. | 43 | 10.2% |
| I was admitted to a hospital due to symptoms of Long COVID. | 8 | 1.9% |
| I have experienced Long COVID symptoms for three months or longer, which continue to affect me to this day. | 36 | 8.5% |
| I have not experienced Long-COVID. | 336 | 79.4% |

| 4. Were you actively involved in patient care during the COVID-19 pandemic? | | |
| --- | --- | --- |
|  | N | % |
| Yes | 326 | 77.1% |
| No | 97 | 22.9% |

| 5. Were you involved in intensive care of patients with a COVID-19 infection? | | | |
| --- | --- | --- | --- |
|  | | N | % |
| Yes | | 86 | 20.3% |
| No | | 240 | 56.7% |
| Exclusion due to question logic |  | 97 | 22.9% |
